# Effectiveness and safety profile of mesenchymal stem cell secretome as a treatment for severe cases of COVID-19: a randomized controlled trial

**DOI:** 10.12688/f1000research.75580.1

**Published:** 2022-02-03

**Authors:** Murdani Abdullah, Jeanne Adiwinata Pawitan, Cosphiadi Irawan, Rahyussalim -, Dita Aditianingsih, Isabella Kurnia Liem, Robert Sinto, Adityo Susilo, Mira Yulianti, Raden Rara Diah Handayani, Irandi Putra Pratomo, Erlina Burhan, Triya Damayanti, Heri Wibowo, Ismail Hadisoebroto Dilogo

**Affiliations:** 1 Division of Gastroenterology, Department of Internal Medicine, Faculty of Medicine, University of Indonesia, Cipto Mangunkusumo Hospital, Jakarta, 10430, Indonesia; 2 Departement of Histology, University of Indonesia, Jakarta, Indonesia; 3 Division of Hematology and Medical Oncology, Department of Internal Medicine, Faculty of Medicine, University of Indonesia, Cipto Mangunkusumo Hospital, Jakarta, 10430, Indonesia; 4 Department of Orthopaedic and Traumatology, Faculty of Medicine, University of Indonesia, Cipto Mangunkusumo Hospital, Jakarta, 10430, Indonesia; 5 Department of Anesthesiology and Intensive Care, University of Indonesia, Jakarta, 10430, Indonesia; 6 Stem Cell Medical Technology Integrated Service Unit, Faculty of Medicine, University of Indonesia, Jakarta, 10430, Indonesia; 7 Department of Anatomy, University of Indonesia, Jakarta, Indonesia; 8 Division of Tropical Medicine and Infectious Diseases, Department of Internal Medicine, Faculty of Medicine, University of Indonesia,Cipto Mangunkusumo Hospital, Jakarta, Indonesia; 9 Division of Pulmonology, Departement of Internal Medicine, Faculty of Medicine, University of Indonesia, Cipto Mangunkusumo Hospital, Jakarta, Indonesia; 10 Department of Pulmonology and Respiratory Medicine, Universitas Indonesia Hospital, Depok, Indonesia; 11 Department of Pulmonology and Respiratory Medicine, Faculty of Medicine, Universitas Indonesia, Persahabatan General Hospital, Jakarta, 10430, Indonesia; 12 Integrated Laboratory, Faculty of Medicine, University of Indonesia, Jakarta, Indonesia

**Keywords:** COVID-19, secretome, mesenchymal stem cell, cytokine, inflammation mediators

## Abstract

**Background:** : Patients with severe COVID-19 had a higher increase in pro-inflammatory and anti-inflammatory cytokines than patients with moderate COVID-19. Excessive release of cytokine and chemokines can lead to multi-organ failure, increasing disease severity, length of stay, and mortality rate. Mesenchymal stem cells (MSCs) are known to have immunomodulatory, anti-inflammatory, anti-apoptotic, and angiogenesis effects that are useful for relieving inflammation, recovery, and protection of lung tissues in COVID-19 patients. Secretome, a secretory product of MSCs, has several advantages over MSCs. Therefore, we conducted a study to investigate secretomes’ effectiveness and safety profile as a treatment for severe COVID-19.

**Methods:** This study was a double-blind, multicentered, randomized, placebo-controlled trial. This study involved 40 subjects recruited from three top COVID-19 referral hospitals in the Greater Jakarta area, Indonesia. Eligible subjects (n=40) were randomized in a 1:1 ratio to intervention group (n=20) and a control group (n=20). The primary outcome of this study was the improvement of inflammatory markers levels, measured by changes in inflammatory markers, and ratio of inflammatory to anti-inflammatory markers. The secondary outcomes of this study included clinical outcome, laboratory outcome, radiological outcome, RT-PCR result conversion, and safety profile of MSC secretome.

**Results:** IL-6 marker in the control group was increased on the 14 ^th^ day after the intervention compared to before intervention [4.110 (2.403–12.820) at baseline to 13.320 (2.958–33.285) on 14 ^th^ day after intervention, p=0.017]. In the intervention group, there was no increase in the IL-6/IL-10 ratio. In contrast, in the control group, there was a significant increase in the IL-6/IL-10 ratio (p=0.036) on the 14 ^th^ day after the intervention compared to before the intervention. We also found that on the seventh day after the intervention, most of the subjects who received placebo had high levels of IL-6 and ferritin (p=0.043). There was no significant difference in the laboratory outcome, radiological outcome, RT-PCR result conversion, and safety profile between both groups.

**Conclusions:** Our study showed an increase of inflammation markers in the control group on the 14 ^th^ day after the intervention, compared to the intervention group. The ratio of inflammatory to anti-inflammatory markers on the seventh and 14 ^th^ days after intervention also did not increase in the intervention group. On the seventh day after intervention, most of the subjects in the control group also had high IL-6 levels and high ferritin levels. There is no adverse event reported. MSC secretome is a safe and promising treatment modality for severe COVID-19.

## Background

The spread of severe acute respiratory syndrome coronavirus 2 (SARS-CoV-2) infection has caused a global COVID-19 pandemic. ^1^ The severity of COVID-19 varies from mild to severe. ^2^ The clinical symptoms of mild and moderate COVID-19 patients, include fever, myalgia, fatigue, and dyspnea, whereas acute respiratory distress syndrome (ARDS) and multiple organ failure usually develop in severe COVID-19 patients. ^2^^,^^3^ Multiple organ failure in COVID-19 can be induced by SARS-CoV-2 infection to target organs expressing ACE2 receptor or by the cytokine storms. ^3^^,^^4^

Increased levels of inflammatory mediators in the blood of COVID-19 patients are related to the severity of COVID-19. ^5^^,^^6^ Patients with severe or critical COVID-19 have immune response dysfunction. ^7^ Patients with severe COVID-19 have a higher increase in pro-inflammatory and anti-inflammatory cytokines than patients with moderate COVID-19. ^5^^,^^6^ Kong *et al.* (2020) in their study stated that there was an increase in levels of VEGF, TNF-alpha, SCF, LIF, IL-2, IL-4, IL-6, IL-8, IL-10, IL -15, IL-17A, IL-18, IL-1beta, and IFN-gamma in severe and critical COVID-19 patients. ^8^ An excessive immune response and overproduction of inflammatory cytokine lead to cytokine storms which induce cytokine release syndrome (CRS). ^2^^,^^9^ Excessive release of cytokine and chemokines in cytokine release syndrome (CRS) can lead to multi-organ failure, which increases the disease severity, length of stay, and mortality rate. ^3^^,^^9^

Currently, there is no definitive therapy used to treat COVID-19 and its immunologic complications. ^2^^,^^10^ Immunosuppressive and immunomodulatory agent is considered as a treatment of immunologic complications in COVID-19. ^10^ Mesenchymal stem cells (MSCs) are known to have immunomodulatory, anti-inflammatory, anti-apoptotic, and angiogenesis effects that are useful for relieving inflammation, recovery, and protection of lung tissues in COVID-19 patient. ^4^^,^^11^ Several studies suggest that mesenchymal stem cells are useful in the treatment of COVID-19. ^12^^,^^13^ A clinical trial in Indonesia showed that COVID-19 patients who received MSCs therapy had a greater decrease in the pro-inflammatory cytokine IL-6 and ferritin compared to patients who did not receive MSCs therapy. In addition, the administration of MSCs therapy was also beneficial in increasing the survival rate of COVID-19 patients. ^12^ Another study stated that COVID-19 patients had PaO _2_/FiO _2_ ratio and radiological improvement after the administration of UC-MSCs. ^13^

MSC are known to secrete bioactive compound that have the same effects as MSC, called secretome. ^11^ A case series on the use of MSC secretome in patients with severe COVID-19, reported that 3 patients with severe COVID-19 had clinical, radiological, and laboratory improvements after the administration of MSC secretome. ^14^ In addition, the use of MSC secretome as a cell-free therapy has wide advantages compared to MSC as cell-based therapy. ^11^^,^^15^ MSC secretome has less tumorigenicity effect, less immunogenicity effect, low risk of emboli formation, and low risk of infection transmission. ^4^^,^^16^ Other advantages of MSC secretome as cell-free COVID-19 therapy, include cost-effective, can be produced in large quantities, ready to used, easy storage with maintaining product potency, and feasibleness in clinical practice. ^4^^,^^11^^,^^16^ Thus, MSC secretome is a new strategy for the treatment of COVID-19. ^17^ Therefore, we conducted a study to investigate the effectiveness and safety profile of MSC secretomes as a treatment for severe COVID-19.

## Methods

### Study design

This study was a double-blind, multicenter, randomized, placebo-controlled trial of MSC secretome in severe COVID-19 patients.

### Participants

The subjects who participated in this study were 40 subjects. The subjects were recruited from Dr. Cipto Mangunkusumo Hospital, Universitas Indonesia Hospital, and Persahabatan National Respiratory Hospital, in the Greater Jakarta area, Indonesia. The recruitment was conducted from February 2021 until July 2021. The subjects who participated in this study had met the inclusion and exclusion criteria. The inclusion criteria were 1) All individuals aged 18–65 years old, 2) Confirmed positive for COVID-19 based on real-time reverse-transcription polymerase chain reaction (RT-PCR) examination on throat/sputum/bronchoalveolar lavage (BAL) swab specimen, 3) Categorized as severe or moderate-to-severe cases of COVID-19 patients, 4) Not intubated on admission, 5) Patients were given standard COVID-19 therapy, 6) Agree to participate and sign the informed consent. Severe COVID-19 was described according to the national guideline released by the Indonesian Ministry of Health, complying with the one released by WHO. ^18^ Moderate to severe COVID-19 criteria was described by COVID-19 pneumonia along with the presence of at least one of the following: sequential organ failure assessment (SOFA) score ≥5.65, body mass index ≥35, D-dimer >1 μg/mL, prothrombin time >13.6 seconds, thrombocytopenia (<100000/mL), INR >1.8, C-reactive protein >25, procalcitonin >0.5 ng/mL, lymphopenia (<1.5×10 ^9^/L), neutrophil-lymphocyte ratio >3.3, or classified as a highrisk group [aged 60 years old, have comorbidity (diabetes, hypertension, lung diseases, and/or asthma), and/or long-term use of steroid]. The exclusion criteria were 1) a pre-existing history of allergy to penicillin, streptomycin, and amphotericin B, 2) a pre-existing cancerous illness, 3) currently participating in other intervention studies, 4) had participated in other intervention studies within the last three months.

### Randomization and masking

Eligible subjects (n=40) were randomized in a 1:1 ratio to receive MSC secretome in the intervention group (n=20) or to receive placebo in the control group (n=20). Subjects were randomized with blocks, block sizes of 4, and two groups (intervention and control) using a randomization software ( Sealed Envelope) by an independent statistician. Study preparations were prepared by a third party who had no involvement with patient care and data collection. All investigators, treating clinicians, and subjects were blinded.

### MSC secretome product preparation

MSC secretome was produced and supplied by Stem Cells Medical Technology, Integrated Service Unit of Faculty of Medicine, Universitas Indonesia – Dr. Cipto Mangunkusumo, Jakarta, Indonesia. The secretome used in this study was derived from the fifth passage of the umbilical cord MSCs. The secretome has passed the quality control stage, sterility, and protein content test.

### Intervention

Prior to the intervention, examination of clinical symptoms and vital signs, laboratory test, inflammation marker test, chest radiograph, and RT-PCR for SARS-CoV-2 were performed on subjects. Subjects in the intervention group (n=20) received MSC secretome and COVID-19 standard therapy. Meanwhile, subjects in the control group (n=20) received placebo (NaCl 0.9%) and COVID-19 standard therapy. MSC secretome was given once at a dose of 15 mL per administration dissolved in 100 mL of normal saline. Secretome was given intravenously for 60 minutes. COVID-19 standard therapy given to both groups were based on the national protocol for COVID-19 therapy. ^18^

### Outcomes

Primary outcomes

The primary outcome of this study was the effect of mesenchymal stem cell secretome on the level of inflammatory markers. The inflammatory markers assessed in this study were interleukin 6 (IL-6), interleukin 10 (IL-10), leukemia inhibitory factor (LIF), vascular endothelial growth factor (VEGF), and ferritin. Examination of inflammatory markers was performed before the intervention and repeated on the seventh and 14 days after the intervention. Parameters analyzed were changes in inflammatory markers and the ratio of inflammatory to anti-inflammatory markers.

Secondary outcomes

The secondary outcomes of this study included clinical outcome, laboratory outcome, radiological outcome, RT-PCR result conversion, and safety profile of MSC secretome. Laboratory parameters, radiological imaging, RT-PCR examination for SARS-CoV-2 data before and after intervention (maximum 14 days after the intervention) were obtained. Side effects, infusion reaction, allergic reaction, secondary infection, adverse event, and renal function test were assessed during hospitalization and maximum of 14 days after the intervention.

### Sample collection

Inflammatory markers were assessed using peripheral venous whole blood samples. Whole blood samples were collected on day 0 (immediately before intervention), on the seventh day after intervention, and on the 14 ^th^ day after intervention. After collection, vacuum tubes were inserted into primary (zip-lock plastic), secondary (thermos), and tertiary pack (cool box containing ice pack) for transportation.

### Statistical analysis

All data in this study were analyzed using SPSS software, V.25.0 (IBM). Analyses of categorical variables were calculated using the Chi-squared test or using Fisher’s exact test. Analyses of unpaired continuous variables were calculated using independent-samples Mann–Whitney U test for non-normally distributed data or independent-samples T-test for normally distributed data. Analyses of paired continuous variables were calculated using paired T-test for normally distributed data or Wilcoxon test for non-normally distributed data.

## Results

### Recruitment

**Figure 1.**
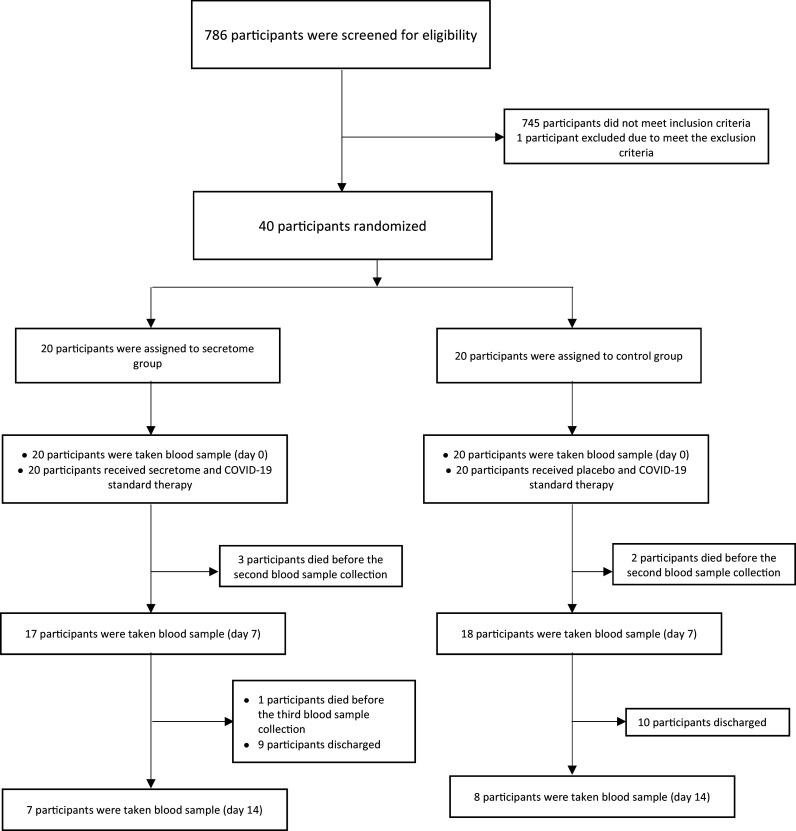
Subject recruitment.

From 18 February 2021 to 9 July 2021, 786 were screened for eligibility. The patient screening was carried out in three hospitals in Jakarta Greater area, Indonesia. A total of 745 patients who did not meet the inclusion criteria were determined ineligible. There was one patient who met the inclusion criteria but could not participate in this study due to being intubated before the intervention. 40 eligible subjects were randomized into two groups in a 1:1 ratio. 20 subjects in the intervention group receive secretome – MSC and COVID-19 standard therapy. Meanwhile, 20 subjects in the control group received placebo and COVID-19 standard therapy. After the intervention, whole blood samples were collected two times (on the seventh day and 14 ^th^ day after intervention) to assess the inflammation markers’ level. The recruitment of the subjects is described in Figure 1.

### Baseline characteristics

This study analyzed 40 subjects and assigned 20 subjects to the intervention group and 20 subjects to the control group. There were no significant differences in the baseline characteristics of the subjects between the intervention and control groups. The average age of subjects in the intervention group was 51.25 years old, whereas the average age of subjects in the control group was 49.9 years old. Most of the subjects in both groups were 40–60 years old. The majority of subjects in both groups were obese. Besides obesity, heart disease was the most common comorbidity in the intervention group. Meanwhile, in the control group, hypertension became the most common comorbidity after obesity. A total of seven subjects in the intervention group had ≥4 comorbidities, while the number of subjects in the control group who had ≥4 comorbidities was 1 (5%) subject. Characteristics of the subject are presented in Table 1.

**Table 1.** Baseline characteristics of severe COVID-19 patient.

Characteristics	Intervention group (n=20)	Placebo group (n=20)	P-value
Age, year (mean±SD)	51.25±(10.51)	49.9±(13.15)	0.722 ****
<40 years, n (%)	3 (7.5)	4 (10)	1.00 ***
40–60 years, n (%)	13 (32.5)	11 (27.5)	
>60 years, n (%)	4 (10)	5 (12.)	
Sex			
Male, n (%)	11 (27.5)	11 (27.5)	1.00 *
Female, n (%)	9 (22.5)	9 (22.5)	
Smoking			
Never, n (%)	16 (40)	16 (40)	1.00 *
Active smokers, n (%)	1 (2.5)	1 (2.5)	
Ex-smokers, n (%)	3 (7.5)	3 (7.5)	
Physical activities			
Almost never, n (%)	15 (75)	10 (25)	0.248 *
Lack of exercise, n (%)	3 (7.5)	5 (12.5)	
Sufficient exercise, n (%)	2 (5)	5 (12.5)	
Number of comorbidity			
0-1	5 (12.5)	9 (22.5)	0.056 ***
2-3	8 (20)	10 (25)	
≥ 4	7 (17.5)	1 (2.5)	
Comorbidity			
Obesity	15 (37.5)	13 (32.5)	0.490 *
Diabetes mellitus	9 (22.5)	6 (15)	0.327 *
Heart disease	11 (27.5)	6 (15)	0.110 *
Hypertension	9 (22.5)	7 (17.5)	0.519 *
Dyslipidemia	0 (0)	1 (2.5)	1.00 **
Encephalitis	1 (2.5)	0 (0)	1.00 **
Stroke	1 (2.5)	0 (0)	1.00 **
Asthma	1 (2.5)	0 (0)	1.00 **
Tuberculosis	1 (2.5)	1 (2.5)	1.00 **
Stress ulcer	1 (2.5)	0 (0)	1.00 **
Dyspepsia syndrome with alarm sign	0 (0)	1 (2.5)	1.00 **
Kidney disease	3 (7.5)	1 (2.5)	0.605 **
Sepsis	2 (5)	0 (0)	0.487 **
Autoimune	1 (2.5)	1 (2.5)	1.00 **
Adenomyosis	0 (0)	1 (2.5)	1.00 **

Data is presented as counts (percentage) for categorical variables.

* p-values were calculated by Chi-squared test.

** p-values were calculated by Fisher’s exact test because there are cells with expected count < 5.

*** p-values were calculated by Mann–Whitney test.

**** p values were calculated by independent-samples T test for normally distributed data of continuous variables.

### Changes in inflammatory markers

The inflammatory markers level in the intervention and control groups were assessed on day 0 (before intervention), the seventh day, and 14 ^th^ day after intervention. Both groups had a comparable baseline levels of inflammatory markers. On the seventh day after intervention, the median values of IL-6, IL-10, LIF, and ferritin in the intervention group were lower than the control group. Meanwhile, the median value of VEGF in the intervention group was higher than the control group. There were no significant differences in inflammatory markers level on the seventh day and 14 ^th^ day after intervention between both groups. However, the IL-6 marker in the control group was significantly increased on the 14 ^th^ day after intervention (n=8) compared to the IL-6 marker before intervention (n=20) [4.11 (2.4025–12.82) at baseline to 13.32 (2.9575–33.285), p=0.017]. The baseline and after intervention levels of inflammatory markers are presented in Table 2.

**Table 2.** Inflammatory marker level at baseline and seventh day after intervention.

Inflammatory marker	Intervention group		p-values	Control group		p-values
	Baseline (n = 20)	Day 7 (n = 17)		Baseline (n = 20)	Day 7 (n = 18)	
IL-6	3.770 (1.713-8.248)	2.270 (1.495-4.455)	0.605 *	4.110 (2.403-12.820)	2.700 (1.670-8.118)	0.862 *
IL-10	1.670 (1.305-1.990)	1.450 (1.240-1.800)	0.691 *	1.670 (1.240-2.073)	1.615 (1.350-2.018)	0.425 *
LIF	6.770 (5.803-8.040)	6.770 (5.480-8.040)	1.00 *	6.778 (6.770-8.985)	7.405 (6.770-8.040)	0.441 *
VEGF	71.195 (27.138-140.093)	55.160 (32.775-115.615)	0.687 *	72.260 (16.310-124.773)	50.055 (27.570-96.773)	0.616 *
Ferritin	258.500 (112-493)	203 (89.500-401.500)	0.148 **	484 (270.500-714.500)	330.500 (198-552.750)	0.356 **

Data is presented as median (interquartile).

* p-values were calculated using Wilcoxon signed rank test.

** p-values were calculated using paired T-test.

**Figure 2.**
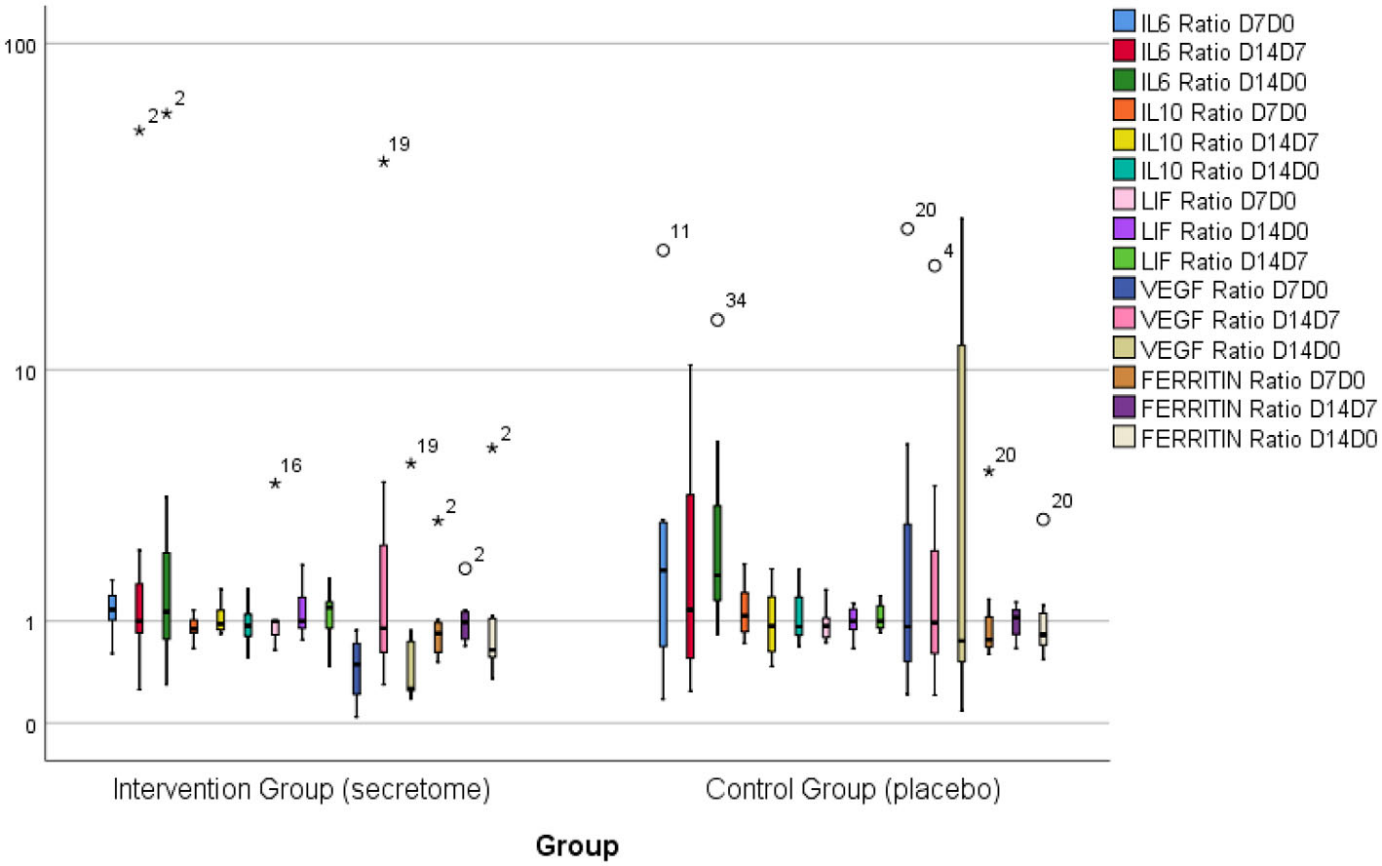
Ratio of inflammation marker level for each time point. Note that the ratios of inflammation markers on IL-6, IL-10, and VEGF in the control group tend to be more varied. The median ratio of IL-6 and VEGF also tend to be higher in the control group. There is no significant difference between both groups.

The ratio of inflammation marker level for each time point is presented in Figure 2. The ratio of inflammation marker is obtained by dividing subjects’ inflammation marker level at time point 1 by the same inflammation marker level at time point 2. Although the ratio of inflammation marker level for some inflammation markers (IL-6, IL-10, VEGF) in the control group tend to be more varied and have higher medians, there were no significant differences in the inflammation marker level ratio on the seventh day to day 0, 14 ^th^ day to the seventh day, and 14 ^th^ day to day 0 between both groups.

**Figure 3.**
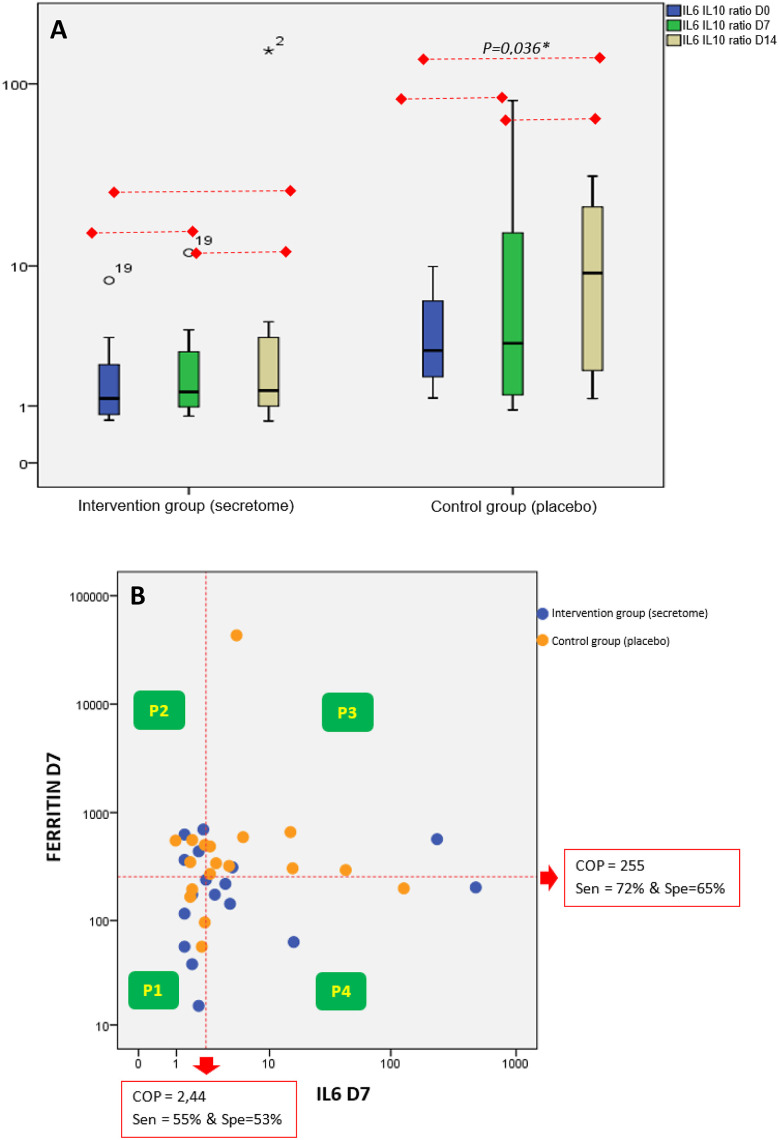
Analyses of IL-6, IL-10, and Ferritin. A) Ratio of IL-6/IL-10 on Day 0 (Before Intervention), the seventh day, and the 14 ^th^ day After Intervention. The IL-6/IL-10 ratio is increasing within 14 days after intervention in the control group. B) Relationship Between IL-6 and Ferritin on the seventh day After Intervention. Most of the subjects in the control group were distributed in the P-3 (high level of IL-6 and ferritin).

Analysis of pro-inflammatory (IL-6 and ferritin) and anti-inflammatory (IL-10) mediators, is presented in Figure 3. IL-6 and IL-10 is presented in the ratio form ( Figure 3A). The ratio value was made based on the quotient between IL-6 and IL-10. We analyzed the changes in the IL-6/IL-10 ratio on day 0 (before intervention), the seventh day, and 14 ^th^ day after intervention ( Figure 1). In the control group, there was a significant increase in the IL-6/IL-10 ratio (p = 0.036) on the 14 ^th^ day after the intervention compared to before the intervention. In contrast, in the intervention group, there was no increase in the IL-6/IL-10 ratio.

The interaction between high and low ferritin levels and high and low IL-6 levels on the seventh day was shown in the form of a scatter graph categorized into 4 panels ( Figure 3B). The scatter graph shows that the proportion of subjects from the control group is mostly distributed in panel-3 (P-3) where P-3 describes the category of high IL-6 levels and high ferritin levels and is significantly larger than the intervention group (p = 0.043). Meanwhile, in the intervention group, most of the subjects were distributed in panel-1 (category of low IL-6 levels and low ferritin levels) and panel-4 (category of high IL-6 levels and low ferritin levels).

### Secondary outcome

Prior to the intervention, most of the subjects, 10 subjects in the intervention group and 12 subjects in the control group, had oxygen saturation >95% using a high-flow nasal cannula. Meanwhile, after the intervention, 10 subjects in the intervention group and eight subjects in the control group had oxygen saturation >95% at room temperature. In this study, there were four subjects from the intervention group and two subjects from the control group who reported using a ventilator after the intervention. There were no differences in oxygen saturation and supplementation between the intervention and control group (before intervention p=0.640, after intervention p=0.883).

The post-intervention laboratory parameters for the intervention and control groups are shown in Table 3. Both groups had comparable laboratory parameters at baseline. Both groups also had comparable levels of post-intervention laboratory parameters, except chloride levels (p=0.035). There was no significant difference in the post-intervention levels of other laboratory parameters between both groups.

**Table 3.** Post-intervention laboratory parameters.

Laboratory parameters (n secretome/n control)	Intervention group	Control group	p-values
Haemoglobin (16/17)	13.500 (12.150-15.275)	13.400 (11.950-15.150)	0.673 **
Haematocrit (16/17)	37.500 (34.500-43.675)	37.400 (36.300-42.600)	0.908 **
WBC (16/17)	13,190 (9,830-17,832.500)	17,110 (10,760-20,240)	0.402 *
Platelets (16/17)	289,500 (191,000-405,500)	336,000 (204,500-378,500)	0.719 **
Erythrocyte (16/17)	4.580 (4.253-5.125)	4.680 (4.235-5.115)	0.754 **
Basophils (13/15)	0.300 (0.200-0.400)	0.300 (0.200-0.500)	0.453 **
Eosinophils (13/15)	0.100 (0-1.900)	0.100 (0-1.100)	0.717 *
Neutrophils (13/15)	83.300 (64.950-93.300)	87.300 (73.500-91.700)	0.722 **
Lymphocytes (13/15)	9.700 (4.100-18.800)	7.400 (4.600-16.300)	0.949 **
Monocytes (13/15)	5.900 (2.400-10.700)	4.600 (3-9.400)	0.717 *
Neutrophils Lymphocytes Ratio (7/10)	14.090 (8.090-50.580)	13.195 (5.723-23.458)	0.740 *
Ureum (12/12)	57.450 (42.175-77.200)	46 (36.500-98.375)	0.478 *
Creatinine (12/12)	0.880 (0.713-1.208)	0.700 (0.600-0.923)	0.265 **
AST (14/16)	29.500 (21.750-55.250)	29 (23.500-40)	0.822 *
ALT (14/16)	54 (38.250-89.750)	61 (37-91)	0.637 *
Procalcitonin (14/12)	0.200 (0.050-0.958)	0.130 (0.045-0.323)	0.781 *
CRP (16/15)	8.150 (3.025-11.875)	12 (1.500-23.400)	0.740 *
Albumin (4/9)	3,170 (2,773-3,350)	3,030 (2,905-3,305)	0.935 **
GFR (12/12)	92.350 (70.550-110.475)	98.400 (84.100-131.850)	0.369 **
D-dimer (14/17)	1,765 (581.203-4,560)	1,500 (488.265-4,805)	0.891 *
Sodium (14/14)	138.500 (135.750-140.250)	136 (132.750-139.250)	0.770 **
Potassium (14/14)	4.110 (3.778-4.493)	4.205 (3.660-4.685)	0.734 *
Chlorida (14/14)	105.250 (102.825-106.850)	102.600 (100.925-103.800)	0,035 *

Data is presented as median (Interquartile).

* p-values were calculated by Mann–Whitney test for non-normally distributed data of continuous variables.

** p values were calculated by independent-samples T test for normally distributed data of continuous variables.

Conversion of RT-PCR results for SARS-CoV-2, from positive to negative, occurred in 8 subjects in the intervention group and 11 subjects in the control group. RT-PCR results of two subjects in the intervention group and seven subjects in the control group converted to negative within ≤14 days. Meanwhile, RT-PCR results for the other six subjects in the intervention group and four subjects in the control group converted to negative within >14 days. There were no significant differences in the number of RT-PCR conversions between the intervention group and control groups (p=0.238).

**Figure 4.**
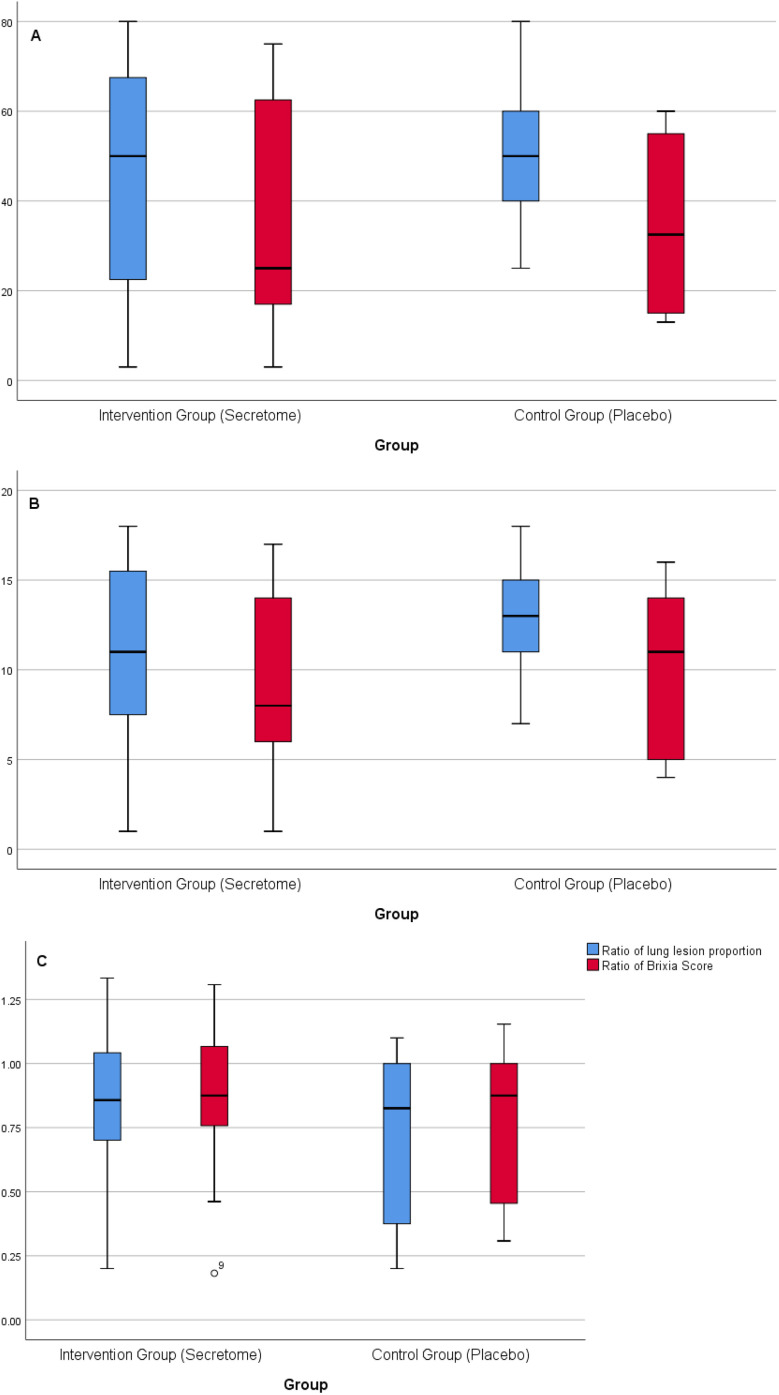
Analyses of chest radiography parameters. A) The proportions of lung lesion area before and after intervention in the intervention and control groups. Blue boxes represent proportions of lung lesion area before intervention, and res boxes represents proportions of lung lesion area after intervention. The median proportions of lung lesion area after intervention was decreased in both groups (deeper in intervention group), but not statistically significant. B) The chest radiography score (Brixia score) before and after intervention in the intervention and control groups. Blue boxes represent Brixia score before intervention and red boxes represents Brixia score after intervention. The median Brixia score after intervention was decreased in both groups (deeper in intervention group), but not statistically significant. C) The ratio of lung lesion area proportion and chest radiography score, after the intervention to before the intervention.

The proportions of lung lesion area and chest radiography score before and after intervention in the intervention and control groups are presented in Figure 4A and B. The intervention and control groups had comparable baseline values for lung lesion area proportions and brixia scores. There were no significant differences in the median value of lung lesion area proportion and brixia scores between both groups before the intervention. After the intervention, the median value of the lung lesion area proportion and brixia score in the intervention group was lower than in the control group. There were no significant differences in the median value of lung lesion area proportion and brixia scores between both groups after the intervention. We analyzed the ratio of lung lesion area proportion and chest radiography score after the intervention to before the intervention. The lung lesion area proportion ratio was made based on the quotient between lung lesion area proportion after the intervention and before the intervention. Meanwhile, brixia score ratio was made based on the quotient between brixia score after the intervention and before the intervention. The ratios oflung lesion area proportion and brixia score after the intervention to before the intervention are presented in Figure 4C. After the intervention, there was a decrease in the lung lesion area proportion and brixia score, both in the intervention and control groups. There were no significant differences in both groups.

A total of seven deaths were reported in this study, four deaths from the intervention group and three deaths from the control group. Six deaths occurred during the study period (within 14 days after intervention). Meanwhile, one death from the control group occurred near to study observation period (18 ^th^ day after intervention). There were no significant differences in the number of deaths between the intervention and control groups (4 *versus* 3, p=1.00). The length of illness in this study was calculated from the onset of symptoms of COVID-19 until the patient recovers. The median value of the length of illness in the intervention group was lower than the control group, but not statistically significant [19 (14.25–23.75) *versus* 21.5 (19.25–27,25), p=0.068, respectively].

## Discussion

Based on the previous studies, MSCs are known to inhibit the release of inflammatory mediators and exert immunosuppressive effects through immune system regulation. ^19^^,^^20^ Saleh *et al.* (2021) conducted a study on five severe COVID-19 patients who were treated by Wharton's jelly-derived mesenchymal stem cells, and the results were a decrease in IL-6 and VEGF levels and an increase in IL-10 levels after the intervention. ^21^ Another study conducted by Adas *et al.* (2021) stated that there were a decrease in IL-6 and ferritin levels and an increase in VEGF levels on the seventh day after the intervention with Wharton's jelly-derived mesenchymal stem cells in critically ill COVID-19 patients. ^22^

Analysis of IL-6 levels in our study showed that the median value of IL-6 in the intervention group was lower than the control group, both on the seventh day and 14 ^th^ day after the intervention. The IL-6 marker in the control group was significantly increased on the 14 ^th^ day after the intervention compared to the IL-6 marker before intervention (p=0.017). Meanwhile, analysis of ferritin levels in our study showed that the median value of ferritin on the seventh day and 14 ^th^ day after intervention in the intervention group was lower than in the control group. Ferritin levels on the seventh day and 14 ^th^ day after the intervention were decreased compared to ferritin levels before the intervention, both in the secretome and control groups. However, there was no significant difference between both groups. In our study, it was also found that on the seventh day after the intervention, most of the subjects who received placebo had high levels of IL-6 and ferritin (p=0.043). These results indicate that mesenchymal stem cell secretomes have a role in suppressing the inflammatory process in severe COVID-19. IL-6 is known as a pro-inflammatory cytokine produced in the blood by leukocytes and can be produced in injured tissue by endothelial cells, fibroblasts, or alveolar epithelial cells. Merza *et al.* (2021) in their study hypothesized that increased levels of IL-6 in severe COVID-19 patients were caused by alveolar epithelial cells inflammatory response due to alveolar epithelial cells injury by SARS-CoV-2. ^6^ In addition, ferritin was a marker of systemic inflammation and can be used to determine the prognosis of COVID-19 patients. ^5^^,^^23^ Chen *et al*. (2020) in their study stated that severe COVID-19 patients had higher ferritin levels than moderate COVID-19 patients, indicating the inflammatory process was higher in severe COVID-19 patients. ^5^ Many previous studies have shown that administration of mesenchymal stem cells is beneficial in reducing levels of inflammatory markers, such as IL-6 and ferritin. ^20^^,^^21^

Analysis of IL-10 levels in our study showed that the median value of IL-10 on the seventh day after the intervention in the intervention group was lower than in the control group, but there was no significant difference between both groups. In addition, there was a decrease in IL-10 levels on the 14th day after the intervention compared to IL-10 levels before the intervention in the secretome and control groups, and there was no significant difference between both groups. Our study also analyzed the changes in the IL-6/IL-10 ratio on day 0 (before intervention), the seventh day, and 14 ^th^ day after intervention ( Figure 2). The IL-6/IL-10 ratio describes the inflammatory potential in COVID-19. A high level of IL-6/IL-10 ratio indicated an increase of inflammatory potential. In the control group, there was a significant increase in the IL-6/IL-10 ratio (p = 0.036) on the 14 ^th^ day after the intervention compared to before the intervention. In contrast, in the intervention group, there was no increase in the IL-6/IL-10 ratio. These results indicate that the administration of secretome is able to control the inflammation in COVID-19. IL-10 is known as an anti-inflammatory cytokine that can suppress inflammatory reaction. ^6^ Chen *et al.* (2020) in their study stated that in most cases of severe COVID-19, there was an increase level of proinflammatory cytokines (IL-6) and anti-inflammatory cytokines (IL-10), and the levels of IL-6 and IL-10 in severe COVID-19 patients were significantly higher compared to moderate COVID-19 patients. ^5^ This indicates the relationship between the severity of COVID-19 and the incidence of cytokine storms in COVID-19. Mesenchymal stem cell therapy from the umbilical cord in severe and critical COVID-19 patients has an effect on the profile of proinflammatory and anti-inflammatory cytokines. Zhang *et al.* (2021) in their case report stated that there was a significant downward trend in IL-6 and IL-10 after infusion of hUCMSC at a dose of 6.4×10 ^7^ twice in critically ill COVID-19 patients. ^24^ This supports the results of our study.

Analysis of VEGF levels in our study showed that there was a decrease in VEGF levels on the seventh and 14 ^th^ days after the intervention compared to before the intervention, both in the secretome and control groups and there was no significant difference between both groups. In addition, the median value of VEGF on the 14 ^th^ day after intervention in the intervention group was lower than in the control group, but there was no statistically significant difference between both groups. VEGF is one of the growth factors associated with the severity of COVID-19. VEGF levels were found to be significantly higher in critically ill COVID-19 patients compared to severe patients. ^8^ Elevated VEGF levels are common in acute inflammatory and hypoxic conditions. Saleh *et al.* (2021) in their study stated that there was a gradual decrease in VEGF levels after Wharton’s Jelly – MSCs administration. ^21^ These results were appropriate with the results in our study.

Analysis of LIF levels in our study showed that the median value of LIF in the intervention group on the seventh day and 14th day after the intervention was lower than in the control group. In addition, LIF levels on the seventh day and 14th day after the intervention were relatively the same as LIF levels before the intervention, both in the secretome and control groups. LIF is an inflammatory mediator that protects the lung during pneumonia. LIF is required to repair and regenerate alveolar epithelial cells during pneumonia due to COVID-19. LIF also prevents vascular leakage due to the inflammatory process. ^25^ Quinton *et al. (*2012) in their study stated that low levels of LIF during pneumonia can cause lung injury. The results of their study stated that the administration of anti-LIF caused an increase in the ratio of the wet lung to the dry lung compared to the control group. ^26^ Study conducted by Dilogo *et al. (*2021) stated that there was an increase in LIF in most subjects who received MSC on the seventh day after the intervention. ^12^

The severity of COVID-19 varies, ranging from asymptomatic, mild symptoms, severe symptoms with respiratory failure and death. ^27^ In our study, 7 (17.5%) deaths were reported, 4 (10%) deaths occurred in the intervention group and 3 (7.5%) deaths occurred in the control group. Six deaths occurred during the study period (within 14 days after intervention). Meanwhile, one death from the control group occurred near the study's observation period (died on day 18 after the intervention). There was no significant difference in the number of deaths in the intervention group, compared with the control group. The mortality rate in our study was lower than in the other study. Dilogo *et al. (*2021) in their study in critically ill COVID-19 patients stated that 10 (25%) deaths occurred in the MSC group and 16 (45%) deaths occurred in the control group. ^12^ Another study in critically ill COVID-19 patients conducted by Adas *et al. (*2021) stated that there were three deaths (30%) in the MSC group and six deaths (60%) in the control group. ^22^

In our study, we reported that in the intervention group there were two subjects who died had ≥4 comorbidities (10%), one subject who died had three comorbidities (5%), and one other subject had one comorbidity (5%). Meanwhile, in the control group, one subject who died had two comorbidities (5%), one subject who died had one comorbid (5%), and one other subject had no comorbid (5%). The comorbidities have a role in the mortality of COVID-19 patients. Wang *et al. *(2020) in a retrospective observational study stated that there were 116 patients who died from a total of 293 COVID-19 patients. Wang *et al.* (2020) also stated that most death cases in COVID-19 occurred in COVID-19 patients who had comorbidities. The study stated that 44 (37.9%) COVID-19 patients who died had ≥2 comorbidities and 36 (31%) of COVID-19 patients who died had one comorbid. ^27^ Another study conducted by Dilogo *et al.* (2021) also stated that in both the MSC group and the control group, most deaths cases in COVID-19 critically ill patients occurred among patients who had ≥2 comorbids. ^12^ This shows that there is a relationship between the number of comorbidities and mortality in COVID-19 patients. The difference in the number of comorbidities between the intervention and control groups is a factor that needs to be considered in determining the success of secretome therapy in this study.

This study has several limitations. The limited number of subjects in this study is one of the factors that can affect the insignificance of the results in this study. In addition, the number of doses, frequency of administration, and time of administration of secretome are also factors that need to be studied further to determine the efficacy of secretome therapy. The results of this study are also expected to be the basis for further studies regarding the implementation of the use of mesenchymal stem cell secretomes as a COVID-19 therapy.

## Conclusions

Our study showed a significant increase of inflammation markers in the control group on the 14 ^th^ day after the intervention, compared to the intervention group. The ratio of inflammatory to anti-inflammatory markers on the seventh and 14 ^th^ days after intervention also did not increase in the intervention group. On the seventh day after intervention, most of the subjects in the control group also had significantly high IL-6 levels and high ferritin levels. There is no adverse event reported. MSC secretome is a safe and promising treatment modality for severe COVID-19.

## Declarations

### Ethics approval and consent to participate

This study has received ethical approval from The Ethics Committee of the Faculty of Medicine, University of Indonesia – Dr. Cipto Mangunkusumo Hospital with protocol number 20-07-0811 on 10 ^th^ August 2020. All study participants or participants’ family have given their written informed consent regarding their participation and data publication in this study.

### Trial Registration

This study was registered in clinicaltrials.gov ( NCT05122234).

## Data availability

### Underlying data

OSF: “Effectiveness and Safety Profile of Mesenchymal Stem Cell Secretomes as a Treatment for Severe Cases of COVID-19”, https://doi.org/10.17605/OSF.IO/NUAVY. ^28^

This project contains the following file:

• Study Raw Data.xlsx

### Reporting guidelines

OSF: CONSORT checklist for ‘Effectiveness and safety profile of mesenchymal stem cell secretomes as a treatment for severe cases of COVID-19: a randomized controlled trial’, https://doi.org/10.17605/OSF.IO/NUAVY. ^28^

Data are available under the licence of the Creative Commons Attribution 4.0 International license (CC-BY 4.0).
