# Supplementary material for: Effectiveness and safety profile of mesenchymal stem cell secretome as a treatment for severe cases of COVID-19: a randomized controlled trial
Source: F1000Res. 2022 Jul 25;11:143. Originally published 2022 Feb 3. [Version 2] doi: 10.12688/f1000research.75580.2 (PMC11809480; doi:10.12688/f1000research.75580.2)
Supplement: Peer review history

# Reviewer report: Reviewer response for version 2

**DOI:** 10.5256/f1000research.122039.r324976

**Published:** 2025-08-20

**Authors:** Fu-Sheng Wang

**Affiliations:** 1 Senior Department of Infectious Diseases, Medical School of Chinese PLA, The Fifth Medical Center of PLA General Hospital, National Clinical Research Center for Infectious Diseases, Beijing, China

This study conducted a multicenter clinical trial on the treatment of severe COVID-19 with mesenchymal stem cell secretome. The results showed that this treatment plan has good safety and can significantly inhibit the increase of inflammatory markers in patients, providing an important reference for clinical practice in related fields. However, there are still some issues worthy of further attention in the following aspects:​

1. Regarding the result of elevated inflammatory markers in the control group in this study, the correlation between this result and the patient's lung injury area, chest X-ray score, and clinical outcomes has not been clarified. The dynamic changes of inflammatory markers are often closely related to disease progression and the degree of tissue damage. If the quantitative relationship between this indicator and the imaging characteristics of lung injury as well as clinical prognosis can be further revealed through correlation analysis, the clinical significance of the results will be greatly enhanced.​
2. In terms of the design details of multicenter enrollment, the study did not specify the specific number of cases in the treatment group and the control group in the three participating hospitals, which may affect the judgment of the consistency and heterogeneity of the research results. Whether the proportion of case distribution in different hospitals is balanced, whether the sample size is sufficient to support subgroup analysis, and the treatment level may act as a confounding factor to affect clinical outcomes.​
3. The analysis of death cases is insufficient. There were 4 deaths in the treatment group and 3 deaths in the control group in the study, but the cause of death and its correlation with treatment have not been elaborated in detail. It is suggested to supplement the clinical data of death cases, such as the time of death, direct cause of death (whether it is related to the studied treatment, whether there are treatment-related adverse reactions), and treatment response before death.​
4. Before the third sample collection, excluding death cases, 9 out of 16 cases in the treatment group were discharged, and 10 out of 18 cases in the control group were discharged. The potential impact of not collecting relevant samples on the result analysis has not been explored, which may lead to sample structure bias and affect the representativeness of subsequent analysis of indicators such as inflammatory markers.

Is the work clearly and accurately presented and does it cite the current literature?

Yes

If applicable, is the statistical analysis and its interpretation appropriate?

Yes

Are all the source data underlying the results available to ensure full reproducibility?

Yes

Is the study design appropriate and is the work technically sound?

Yes

Are the conclusions drawn adequately supported by the results?

Yes

Are sufficient details of methods and analysis provided to allow replication by others?

Partly

Reviewer Expertise:

Immunology; Infectious diseases

I confirm that I have read this submission and believe that I have an appropriate level of expertise to confirm that it is of an acceptable scientific standard, however I have significant reservations, as outlined above.

# Reviewer report: Reviewer response for version 2

**DOI:** 10.5256/f1000research.122039.r349922

**Published:** 2025-02-06

**Authors:** Usha Rani Kandula

**Affiliations:** 1 Department of Community Health Nursing, Cihan University-Erbil, Erbil, Kuridistan Region, Kuridistan Region, Iraq

The study is well-presented, and citing current literature. The randomized controlled design is appropriate and methodologically sound. Detailed methods and statistical analyses are provided for replication. Source data availability ensures reproducibility. Conclusions are well-supported by results, demonstrating the effectiveness and safety of mesenchymal stem cell secretome for severe COVID-19 cases.

Is the work clearly and accurately presented and does it cite the current literature?

Yes

If applicable, is the statistical analysis and its interpretation appropriate?

Yes

Are all the source data underlying the results available to ensure full reproducibility?

Yes

Is the study design appropriate and is the work technically sound?

Yes

Are the conclusions drawn adequately supported by the results?

Yes

Are sufficient details of methods and analysis provided to allow replication by others?

Yes

Reviewer Expertise:

Nursing research and administration

I confirm that I have read this submission and believe that I have an appropriate level of expertise to confirm that it is of an acceptable scientific standard.

# Reviewer report: Reviewer response for version 2

**DOI:** 10.5256/f1000research.122039.r358402

**Published:** 2025-01-24

**Authors:** Mutiara Indah Sari

**Affiliations:** 1 Universitas Sumatera Utara, Medan, North Sumatra, Indonesia

This randomized controlled trial studies the effectiveness and safety profile of mesenchymal stem cell (MSC) secretome on severe COVID-19. The variables examined include IL-6, IL-10, LIF, VEGF and ferritin levels as well as laboratory parameters, RT-PCR conversion rates, chest radiography parameters, and mortality.

 There are a few points that need to be considered:

 - For both the abstract and the main text, consider using a different term from “intervention” for the placebo treatment of the control group to avoid confusion between the control and intervention group.

 - In the abstract, the results section needs to describe more about the effects of secretome on the intervention group, compared to the control group.

 - In the discussion section of the main text, please elaborate on the mechanism of secretome that leads to the different outcome between the intervention group and the control group. As secretome contains numerous molecules, describe how the dominant molecule in the administered secretome affects the overall outcome.

 - In the conclusion section of the main text, as the aim of this study is to investigate the effectiveness and safety profile of MSC secretome as a treatment for severe COVID-19, compare the biomarker levels of the intervention group to the control group.

Is the work clearly and accurately presented and does it cite the current literature?

Partly

If applicable, is the statistical analysis and its interpretation appropriate?

Yes

Are all the source data underlying the results available to ensure full reproducibility?

Yes

Is the study design appropriate and is the work technically sound?

Yes

Are the conclusions drawn adequately supported by the results?

Partly

Are sufficient details of methods and analysis provided to allow replication by others?

Yes

Reviewer Expertise:

Genetics, Immunology, Metabolism

I confirm that I have read this submission and believe that I have an appropriate level of expertise to confirm that it is of an acceptable scientific standard, however I have significant reservations, as outlined above.

# Reviewer report: Reviewer response for version 2

**DOI:** 10.5256/f1000research.122039.r336476

**Published:** 2024-11-19

**Authors:** Chenxiao Wang

**Affiliations:** 1 Tulane University, New Orleans, USA

The study titled *Effectiveness and safety profile of mesenchymal stem cell secretome as a treatment for severe cases of COVID-19* by Abdullah et al. is a randomized, double-blind, multicenter, placebo-controlled trial aimed at assessing the effectiveness and safety of mesenchymal stem cell (MSC) secretome for severe COVID-19 patients. The trial involved 40 participants, divided into an intervention group receiving MSC secretome and a control group receiving a placebo, with both groups also receiving standard COVID-19 therapy. The primary outcome assessed was the change in inflammatory markers, and secondary outcomes included clinical, laboratory, and radiological outcomes, as well as safety and RT-PCR conversion rates.

 The article provides an in-depth analysis of the potential benefits of MSC secretome as a cell-free therapy for severe COVID-19, highlighting its immunomodulatory and anti-inflammatory properties. However, there are a few questions that need to be further discussed.

 1. Minor point: Is the study design appropriate and is the work technically sound?

 Partly. While the study design is appropriate for the research question, employing a randomized, double-blind, and placebo-controlled approach, a more specific explanation on the administration route is needed: The secretome of mesenchymal stem cells (MSCs) is a collection of substances released by MSCs into their surrounding environment and it is believed to be released and work locally in the lung. In this study, the authors chose the i.v. treatment. This needs more explanation and discussion.

 2. Minor point: In the reference No.8, It states, " *Compared to the severe group, critical cases were of significantly older ages and showed higher white blood cell counts and neutrophil counts. Levels of VEGF-D, TNF-α, SCF, LIF, IL-2, IL-4, IL-6, IL-8, IL-10, IL-15, IL-17A, IL-18, IL-1β, and IFN-γ were significantly higher in the critical group than in the severe group.*" In a word, the reference paper shows the difference between two COVID severities rather than COVID and non-COVID patients as the authors cited in the background part. Please revise.

Is the work clearly and accurately presented and does it cite the current literature?

Yes

If applicable, is the statistical analysis and its interpretation appropriate?

I cannot comment. A qualified statistician is required.

Are all the source data underlying the results available to ensure full reproducibility?

Yes

Is the study design appropriate and is the work technically sound?

Partly

Are the conclusions drawn adequately supported by the results?

Yes

Are sufficient details of methods and analysis provided to allow replication by others?

Yes

Reviewer Expertise:

respiratory immunology; preclinical study.

I confirm that I have read this submission and believe that I have an appropriate level of expertise to confirm that it is of an acceptable scientific standard, however I have significant reservations, as outlined above.
